# Post-Operative Non-Septic Leucocytosis

**Published:** 1902-10-11

**Authors:** 


### POST-OPERATIVE NON-SEPTIC LEUCOCYTOSIS.

Much discussion has taken place regarding the diagnostic and prognostic value of an increase in the leucocytes of the blood. On this point the recently published observations of H. M. King[1] are of considerable interest and indicate that surgeons must be careful in accepting mere blood counts as a guide. He shows that an increase of from 5,000 to 10,000 leucocytes per cubic millimetre following operation in from 6 to 36 or even 48 hours is a normal post-operative condition, provided it be not sustained. The maximum leucocytosis usually occurs within the first 12 hours after operation, and is very transient. The leucocytosis in the normal reparative process bears but slight relation to the pulse and temperature. A post-operative leucocytosis of 10,000 or more above the individual normal sustained for more than a few hours may be looked upon with suspicion. The apparent increase in the number of erythrocytes following operation is not caused by an actual increase in the circulating blood.

[1] Amer. Jour., Sept. 1902.

